# The inducible caspase-9 suicide gene system as a “safety switch” to limit on-target, off-tumor toxicities of chimeric antigen receptor T cells

**DOI:** 10.3389/fphar.2014.00235

**Published:** 2014-10-28

**Authors:** Tessa Gargett, Michael P. Brown

**Affiliations:** ^1^Translational Oncology Laboratory, Centre for Cancer Biology, SA Pathology and University of South AustraliaAdelaide, SA, Australia; ^2^Cancer Clinical Trials Unit, Royal Adelaide HospitalAdelaide, SA, Australia; ^3^Discipline of Medicine, University of AdelaideAdelaide, SA, Australia

**Keywords:** chimeric antigen receptor T cells, inducible caspase 9, AP1903, suicide gene, safety switch, cancer immunotherapy

## Abstract

Immune modulation has become a central element in many cancer treatments, and T cells genetically engineered to express chimeric antigen receptors (CAR) may provide a new approach to cancer immunotherapy. Autologous CAR T cells that have been re-directed toward tumor-associated antigens (TAA) have shown promising results in phase 1 clinical trials, with some patients undergoing complete tumor regression. However, this T-cell therapy must carefully balance effective T-cell activation, to ensure antitumor activity, with the potential for uncontrolled activation that may produce immunopathology. An inducible Caspase 9 (iCasp9) “safety switch” offers a solution that allows for the removal of inappropriately activated CAR T cells. The induction of iCasp9 depends on the administration of the small molecule dimerizer drug AP1903 and dimerization results in rapid induction of apoptosis in transduced cells, preferentially killing activated cells expressing high levels of transgene. The iCasp9 gene has been incorporated into vectors for use in preclinical studies and demonstrates effective and reliable suicide gene activity in phase 1 clinical trials. A third-generation CAR incorporating iCasp9 re-directs T cells toward the GD2 TAA. GD2 is over-expressed in melanoma and other malignancies of neural crest origin and the safety and activity of these GD2-iCAR T cells will be investigated in CARPETS and other actively recruiting phase 1 trials.

## INTRODUCTION TO CAR T-CELL IMMUNOTHERAPY FOR CANCER

Tumor initiation and progression is sculpted by host immunity (Shankaran et al., 2001; Fridman et al., 2012). Since recent US FDA approval of the cancer immunotherapeutic agents, sipuleucel-T and ipilimumab, for the treatment of prostate cancer and melanoma, respectively, immunotherapy has joined surgery, radiotherapy, and chemotherapy as a conventional modality of treatment for non-hematologic malignancies. Melanoma has been a particular target of immunotherapy because of its inherent immunogenicity, and early therapeutic approaches such as interleukin-2 (IL-2) and interferon-alpha2b (IFNα_2b_) aimed to stimulate these antitumor immune responses (Mocellin et al., 2010; Rosenberg, 2014). Recently, monoclonal antibodies (mAb) that inhibit immune checkpoint signaling molecules such as CTLA4 (via iplimumab; Hodi et al., 2010; Wolchok et al., 2013), PD1 or PD-L1, (Hamid et al., 2013; Topalian et al., 2014) have shown recruitment of cytotoxic T-cell responses. *Ex vivo* expansion of tumor infiltrating lymphocytes (TIL) also aims to boost the melanoma-specific immune response and has shown impressive results for selected patients (Rosenberg and Dudley, 2004; Besser et al., 2013). While most of these therapies rely on a pre-existing immune response to tumor-associated antigens (TAA), the adoptive transfer of T cells genetically engineered to express novel TAA-specific receptors enables delivery of an antitumor immunotherapy even in the absence of intrinsic tumor-specific immunity (Restifo et al., 2012). The T cells may express either a transgenic TCR or a chimeric antigen receptor (CAR) and of the two types of gene modification, CAR T cells have shown the most promise in clinical trials, with over 51 CAR clinical trials currently registered in the US alone (clinicaltrials.gov).

A first-generation CAR typically comprises an antigen-binding ectodomain of a mAb-derived single chain variable fragment (scFv) and a signaling endodomain from the CD3ζ molecule. Following expression, the CAR re-directs the specificity of the T cell toward the cognate antigen. Unlike TCR, CAR are independent of MHC-restricted antigen presentation, which is a feature of immune evasion by many different cancers (Seliger et al., 1996; Seliger, 2008), and instead can bind directly to TAA, albeit those located at the cell surface. They can also include multiple intracellular signaling domains from costimulatory molecules. For example, second-generation CAR may contain CD3ζ and CD28 signaling domains, while third-generation CAR may contain CD3ζ, CD28 and either OX40 (CD134) or 4-1BB (CD137). T cells expressing these later generation CAR demonstrate enhanced activation and effector function (Pule et al., 2005). Autologous CAR T cells have shown promising objective responses in leukemia and neuroblastoma patients, including some complete and partial regressions (Pule et al., 2008; Porter et al., 2011; Grupp et al., 2013). However, reports of serious adverse events (AEs) in some of these early trials have raised safety concerns about the technology (see **Table 1**).

**Table 1:** Reports of life-threatening and fatal adverse events in CAR T-cell clinical trials.

Trial	Event	Reference
CAIX CAR T cells (first generation)	Grade 3 and 4 transient liver enzyme increases at 1–2 × 10^9^ total cell dose as on-target toxicity related to CAIX expression on bile duct, preventable by pre-treatment with anti-CAIX monoclonal antibody.	Lamers et al. (2006, 2013)
CD19 CAR T cells (second generation)	Tumor lysis syndrome at 3 × 10^8^/kg total cell dose (1.46 × 10^5^/kg CAR T cells), resolved by day 26 after infusion. Severe cytokine-release syndrome, reversible by monoclonal antibody blockade at 1 × 10^8^/kg cell dose (1.2 × 10^7^/kg CAR T cells).	Porter et al. (2011), Grupp et al. (2013)
HER2/neu (ERBB2) CAR T cells (third generation)	Respiratory distress and death at 1 × 10^10^ total cell dose, likely due to CAR T cell localization to the lung and “cytokine storm.”	Morgan et al. (2010)
CD19 CAR T cells (second generation)	Renal failure, “sepsis-syndrome,” elevated cytokine levels and death at 1.2 × 10^7^/kg, possibly due to a combination of sepsis, cyclophosphamide treatment and T-cell transfer.	Brentjens et al. (2010)

## CAR T-CELL-RELATED SAFETY CONCERNS

The major safety concern for CAR T-cell products is the risk of on-target but off-tumor effects resulting from T-cell activation in response to normal tissue expression of the TAA. Similar off-tumor side-effects have been reported for approved mAb therapies such as trastuzumab (anti-HER2), which has cardiotoxicity in some patients due to targeting of HER2-mediated cardiomyocyte survival pathways, and cetuximab (anti-EGFR), which produces skin rashes and mucosal irritation related to EGFR expression on epithelial cells (Perez-Soler et al., 2005; Chien, 2006). In a more recent example, a phase 1 trial of an anti-EphA2 mAb/cytotoxic drug conjugate was discontinued due to serious AEs of clinical bleeding and coagulation probably because of endothelial-cell targeting (Annunziata et al., 2013). Transgenic TCR T cells likewise feature off-tumor side effects including one trial which reported MART-1- and gp100-specific T-cell killing of normal melanocytes, with the majority of patients experiencing widespread erythematous skin rash, transient uveitis or hearing loss, with most symptoms reversed upon local steroid treatment (Johnson et al., 2009). While in another trial, transgenic TCR T cells specific for the colorectal TAA CEA have resulted in severe transient colitis (Parkhurst et al., 2011). Thus there are multiple reports of toxicity by TAA-targeted cell therapies, and TAA-specific CAR T cells may also result in the targeting of healthy tissues.

There is also the theoretical risk of insertional mutagenesis and oncogenic expansion of transduced cells with retroviral-based gene therapy. Indeed, such an event has been reported in a gene-therapy trial for X-linked Severe Combined Immunodeficiency (X-SCID) in which patients received retrovirally transduced CD34^+^ bone marrow progenitor cells and four out of nine patients then developed acute T-leukemia. It is believed that insertional mutagenesis in the *LMO2* proto-oncogene together with subsequent massive proliferation of the transduced CD34^+^ progenitor population in immunodeficient hosts contributed to T-leukemogenesis (Hacein-Bey-Abina et al., 2003, 2010). In contrast, similar AEs have not been observed in trials of adoptive transfer of autologous, polyclonal gene-modified mature T cells in immunocompetent hosts (Heslop et al., 2010; Scholler et al., 2012). However, there is evidence in murine model systems that mono- or oligo-clonal mature T-cell populations are more susceptible to insertional mutagenesis (Newrzela et al., 2008, 2011, 2012). Most CAR T cells to date have been manufactured from polyclonal peripheral blood T cells (Cooper et al., 2006), and even selected CAR T cells remain polyclonal in respect of their endogenous TCR repertoire (Wang et al., 2012). Nevertheless, insertional mutagenesis remains a concern and is another consideration in long-term clinical monitoring, which is a routine part of gene transfer trials and which helps to establish the safety profile of CAR T cells.

In addition, CAR T cells may also produce on-target, on-tumor toxicities more familiar with other cancer therapies. For example, the tumor lysis syndrome (TLS; Yang et al., 1999; Ji et al., 2013), cytokine release syndrome (CRS) and the related macrophage activation syndrome (MAS) that are associated with some chemotherapies and targeted- or immuno-therapies (Suntharalingam et al., 2006; Teachey et al., 2013). Importantly these AEs may occur during the destruction of tumors, and thus even a successful, on-tumor CAR T-cell effect might result in toxicity that requires intervention.

## CAR T-CELL-RELATED SERIOUS ADVERSE EVENTS

Specific examples of these toxicities have been observed in several CAR T-cell clinical trials (see **Table 1**). Interestingly, evident on-target, off-tumor toxicity does not require the incorporation of additional T-cell costimulatory domains into the CAR construct. In a trial of autologous T-cells expressing a first-generation CAR directed toward carbonic anhydrase IX (CAIX) in patients with metastatic renal cell carcinoma, NCI-CTC grade 3 or 4 liver function abnormalities were observed in 4 of 12 patients. This toxicity was subsequently prevented by pre-treatment with an anti-CAIX mAb (Lamers et al., 2006, 2013).

Two fatal AEs have been reported in phase 1 clinical trials of an anti-HER2 CAR (Morgan et al., 2010) and of an anti-CD19 CAR (Park and Brentjens, 2010). In the third-generation (CD28.4-1BB.ζ) anti-HER2 CAR trial, a patient with pulmonary metastases of colorectal cancer received lymphodepleting chemotherapy followed by an intravenous dose of 10^10^ HER2-CAR T cells. Within 4 h, the patient developed acute respiratory distress and died 5 days later with multi-organ failure. The investigators attributed her death to a “cytokine storm” hypothesized to result from T-cell activation on binding of CAR T cells to HER2-expressing bronchial epithelium. In the trial of a second-generation (CD28.ζ) anti-CD19 CAR, patients received lymphodepleting chemotherapy followed by 1.2–3 × 10^7^ CD19-CAR T cells/kg. One patient developed persistent fevers, respiratory distress and progressive acute renal failure, and died 44 h post-infusion. Unlike other patients enrolled in this study, this patient’s serum cytokine levels were abnormally high after the chemotherapy and before the CAR T-cell infusion but no higher after the CAR T-cell infusion, and the patient’s death was thus attributed to undetected sepsis. However, the direct attribution of these CAR-related AEs to on-target effects is confounded by other factors such as high T-cell dose, first-pass through the pulmonary vasculature after intravenous administration, unintended consequences of conditioning treatments, and possible underlying infection.

Although second-generation CARs incorporating the CD28 or the 4-1BB costimulatory domains have generally been safe (Brentjens et al., 2011, 2013; Savoldo et al., 2011), other reports indicate that serious on-target toxicities including TLS and CRS have occurred (Brentjens et al., 2010; Kalos et al., 2011; Porter et al., 2011; Kochenderfer et al., 2012; Grupp et al., 2013). Four of 8 patients receiving second-generation (CD28.ζ)CD19-specific CAR T cells for progressing B-cell malignancies experienced acute and reversible toxicities, the severity of which correlated with serum cytokine levels (Kochenderfer et al., 2012). In a trial in chronic lymphoid leukemia (CLL) patients, one patient developed TLS after infusion of second-generation (4-1BB.ζ)CD19-CAR T cells requiring hospitalization on day 22 and treatment with fluid resuscitation and rasburicase, with symptoms resolving by day 26 (Porter et al., 2011). In a separate trial of CD19-CAR T cells in acute lymphoblastic leukemia (ALL) patients, two patients experienced CRS and for one patient this resulted in hospitalization on day 4 and transfer to intensive care on day 5 after the T-cell infusion (Grupp et al., 2013). The CRS was reversed by anti-cytokine therapy including tocilizumab (anti-IL-6 receptor mAb) and etanercept (decoy TNF receptor). This patient went on to develop a complete remission, while the other patient had a relapse of CD19-negative leukemia after 2 months. High levels of IL-10 and IL-6 were also detected in these patients, which suggests a MAS induced by non-physiologic T-cell activation (Maude et al., 2014).

As a general statement, early clinical investigation of any CAR T cell of novel specificity may result in unpredictable on-target, off-tumor toxicity. Hence, suicide gene technology can provide a valuable “safety switch” in these at-risk clinical scenarios, allowing for the targeted deletion of inappropriately activated CAR T cells. Also, although we have focused on CAR T-cell-related toxicities in this review, it is worth noting that two patient deaths have been reported for each of two different MAGE-A3-specific transgenic TCR T cells, which resulted from either neurologic toxicity when brain-expressed MAGE-A12 was cross-targeted (Morgan et al., 2013) or cardiac toxicity when heart muscle-expressed Titin was cross-targeted (Linette et al., 2013). The incidence of these toxicities, and of tumor burden-related CRS and MAS after CD19-CAR T cell therapy for B-leukemia (Maus et al., 2014), may be ameliorated by a “safety-switch” if the suicide gene system chosen is sufficiently rapid in onset.

## THE iCasp9 SUICIDE GENE

The herpes simplex virus-thymidine kinase (HSV-TK) suicide gene system has long been used in cell therapy investigations as a method for depleting transduced cells in the case of AEs (Recchia et al., 2006; Ciceri et al., 2009), However, major disadvantages attend its use for this purpose. Activation of HSV-TK by ganciclovir is relatively slow, requiring 3 days to have a complete effect *in vitro* (Marin et al., 2012) and although mutant versions of TK have improved drug sensitivity and killing (Willmon et al., 2006) the viral TK gene product has intrinsic immunogenicity that may cause transduced cells to be rejected by the host immune system in immunocompetent individuals (Riddell et al., 1996; Berger et al., 2006). Additionally, if ganciclovir is used to treat CMV infections in immunocompromised recipients of hemopoietic stem transplants then the use of this suicide gene would result in the unwanted deletion of transduced cells (Bonini et al., 2007; Sangiolo et al., 2011).

**FIGURE 1:**
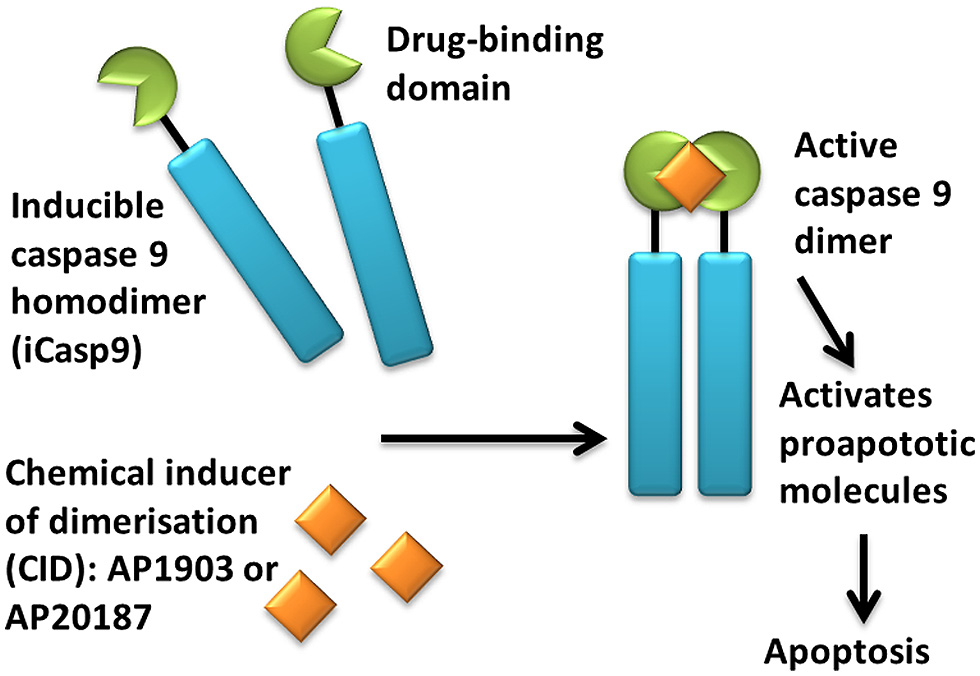
**Activation of iCasp9 results in the death of transduced cells.** Inducible caspase 9 (iCasp9) is produced in the transduced cell as a homodimer with a drug-binding domain. Administration of CID (AP1903 or AP20187) results in dimerization of caspase 9 leading to an activated form of the molecule, which then initiates a signaling cascade leading to apoptosis of the transduced cell.

An alternative suicide gene system is CaspaCIDe®;, which consists of an inducible caspase 9 (iCasp9) gene together with the small-molecule, bio-inert, chemical induction of dimerization (CID) drug, AP1903. The iCasp9 gene contains the intracellular portion of the human caspase 9 protein, a pro-apoptotic molecule, fused to a drug-binding domain derived from human FK506-binding protein (Clackson et al., 1998; Straathof et al., 2005; see **Figure 1**). Intravenous administration of AP1903 produces cross-linking of the drug-binding domains of this chimeric protein, which in turn dimerizes caspase9 and activates the downstream executioner caspase 3 molecule, resulting in cellular apoptosis. The pharmacokinetics of AP1903 has been studied in a placebo-controlled, escalating-dose study in volunteers and the drug has been found to be safe and well tolerated to the highest dose of 1 mg/kg, with plasma levels directly proportional to administered dose and rapid reduction to 1% of the maximum plasma concentration by 10 h (Iuliucci et al., 2001).

In an early preclinical study of this system, it was shown that expression iCasp9 in, retrovirally transduced Epstein Barr virus-specific cytotoxic T lymphocytes (CTLs) in the absence of CID had no effect on the phenotype or function of the transduced cells. However, a single 10 nM dose of CID killed 89–93% of transduced cells by day 7, as measured by GFP expression. These results were confirmed *in vivo* in a SCID mouse-human xenograft model where a single dose killed over 99% of circulating human GFP^+^ T cells by day 3. Importantly, killing *via* iCasp9 has been found to be extremely rapid with early apoptotic Annexin V^+^ cells appearing within 30 min, and a complete effect observed by 24 h of CID treatment *in vitro* (Marin et al., 2012).

Other preclinical studies have demonstrated the feasibility of including retrovirally encoded iCasp9 in cells intended for human therapy. One study investigated the use of iCasp9-transduced donor T cells to reduce the risk of donor T-cell-induced graft-versus-host disease (GvHD) in recipients of a haploidentical stem cell transplant (Tey et al., 2007). Allodepleted donor T cells were transduced with a retroviral vector encoding both iCasp9 and a truncated CD19 molecule as a selectable marker and were shown to retain effector functions after expansion and magnetic selection for the CD19^+^ population. When these donor T cells were activated with allogeneic PBMC, 10 nM CID effectively killed activated cells within 24 h *in vitro* while sparing non-activated virus-specific T cells.

Another study using a mouse model of T-cell mediated tissue destruction has also shown that the iCasp9/CID system can be used to rapidly eliminate T cells and block an ongoing autoimmune attack *in vivo* (de Witte et al., 2008). In this model, iCasp9-transduced, ovalbumin (OVA)-specific OT-I transgenic T cells destroyed OVA-expressing pancreatic β-cells, and led to the rapid induction of diabetes and weight-loss that required euthanasia within 1 week of T-cell administration. However, CID administration removed 85% of transgenic OT-I T cells within 2 days, and significantly inhibited diabetes development so that mice only experienced transient diabetes or remained normo-glycemic. In this study, homeostatic expansion of OT-I T cells was also completely halted by CID administration, which provides support for the use of the iCasp9/CID system in adoptive T-cell therapies provided to lymphodepleted patients.

The first preclinical description of CAR T cells including an iCasp9 gene was reported by Hoyos et al. (2010) who developed second-generation CD19-CAR T cells that co-expressed IL-15 and iCasp9. The authors demonstrated that these iCasp9/CAR.19/IL-15 T cells had improved effector function and were effectively eliminated *in vivo* within 3 days of CID administration, as measured by live imaging of luciferase-positive CAR T cells (Hoyos et al., 2010). A third-generation, anti-CD20 CAR expressing iCasp9 has likewise demonstrated 90% reduction in peripheral blood CAR T cells *in vivo* 12 h after CID administration on two consecutive days (Budde et al., 2013). A study of anti-CD19 CAR T cells co-expressing a modified hygromycin resistance/HSV-TK suicide gene fusion found that transferred T cells did not persist in patients due to anti-transgene immune responses against the resistance or suicide genes, which highlights the need for close clinical monitoring of vector persistence and anti-transgene immunity in clinical trials of T cells expressing iCasp9 (Jensen et al., 2010).

The iCasp9/CID system has been successfully translated to a clinical setting. A trial of donor T-cell transfer in stem cell transplant patients demonstrated that iCasp9 could eliminate transferred T-cells and end GvHD in patients (Di Stasi et al., 2011). Five patients who had received CD34^+^ haploidentical stem cell transplants for relapsed leukemia were given allodepleted donor T-cells transduced to express iCasp9 and the truncated CD19 selectable marker. The sorted, CD19^+^ CD3^+^ transgenic T-cells were 90–93% pure, and were able to survive and expand when transferred to patients. Skin GvHD occurred in four of the five patients and liver GVHD occurred in one patient 14–42 days after T-cell transfer. Treatment of these patients with a single dose of AP1903 (CID) reduced the numbers of circulating transgenic T cells by 90% within 30 min of administration, but had no effect on endogenous T cells and did not cause any AEs. Within 24 h, the GvHD-associated skin abnormalities were terminated and did not return, however, non-alloreactive virus-specific transgenic T cells were subsequently identified in patient blood. A long-term follow up of this clinical study has confirmed that the patients have maintained immune reconstitution, including virus-specific donor T cells, without recurrence of GvHD 3.5 years after the initial T-cell infusion (Zhou et al., 2014). Other trials of iCasp9 gene transfer in haploidentical donor T cells are also ongoing (NCT01494103, ACTRN12614000290695).

An important aspect of the iCasp9/CID system is that it enables the specific killing of highly activated cells with high levels of transgene expression. As retroviral vectors preferentially integrate near transcription start sites and genes involved in proliferation (Wu et al., 2003; Recchia et al., 2006; Cattoglio et al., 2007), it is an inherent feature of the technology that actively dividing cells will have higher transgene expression and hence higher levels of iCasp9. Pre-clinical work suggests that transduced CTLs that are not killed by CID administration express insufficient iCasp9 to allow functional activation by CID (Straathof et al., 2005). However, it has been observed that activation of surviving T cells with down-modulated transgene expression can rapidly restore CID sensitivity (Tey et al., 2007). In the GvHD clinical trial, this incomplete depletion of transduced cells allowed for the re-expansion of the small population of non-alloreactive CD3^+^CD19^+^ T cells remaining after AP1903 treatment. These T cells were rapidly killed when activated and re-exposed to AP1903 *ex vivo,* indicating that the surviving population remained sensitive to AP1903-mediated killing (Di Stasi et al., 2011). Notwithstanding these results, another *in vitro* study has found that immune-selected CTLs remaining after CID treatment have little or no proliferative potential or functional transgene activity. The study also found that immune-selected CTLs rested for 8–9 days after last antigen stimulation can be depleted significantly by CID treatment (Quintarelli et al., 2007). Hence, the functional significance of the iCasp9^+^T cells surviving CID treatment remains an open area of investigation. In the event of AP1903 administration for serious AEs related to either on-target, on-tumor or on-target, off-tumor effects, the persistence and reactivity of transgene-containing CAR T cells will need to be determined *via* studies of patient tissue samples, which may include attempted *ex vivo* re-expansion of transgene-containing T cells.

## FUTURE DIRECTIONS: iCasp9-CAR CLINICAL STUDIES

Autologous iCasp9-expressing CAR T cells are being clinically evaluated in actively recruiting trials. Our imminent phase 1 trial will investigate GD2-specific and iCasp9-expressing CAR (GD2-iCAR) T cells in advanced melanoma patients (CARPETS, ACTRN12613000198729). Autologous patient T cells will be transduced with a retroviral vector encoding a third-generation GD2-specific CD28.OX40.ζ CAR (Pule et al., 2005), which was developed by the Brenner group in a first-generation CAR format for phase 1 clinical evaluation in neuroblastoma patients (Pule et al., 2008; Louis et al., 2011), and then modified further to encode iCasp9 (Gargett et al., 2014). This third-generation GD2-iCAR is also currently being investigated in an open phase 1 trial in neuroblastoma patients (GRAIN, NCT01822652), sarcoma patients (VEGAS, NCT01953900) and other GD2^+^ solid tumors (NCT02107963).

GD2 is a disialoganglioside upregulated on the cell surface of tumors of neuroectodermal origin. The GD2-specific ch14.18 mAb, which has been included in standard treatment regimens for high-risk neuroblastoma (Yu et al., 2010), has the same antigen-binding domain as the 14g2a mAb, which donated the scFv for the GD2-CAR (Pule et al., 2008; Louis et al., 2011). Ch14.18, 14g2a and other GD2-specific mAb can elicit toxicities such as fever, rash, hypotension, and painful peripheral neuropathy (Saleh et al., 1992; Murray et al., 1994), which probably results from complement activation (Sorkin et al., 2010). Although such toxicities are possible in any clinical trial of GD2-CAR T cells, no significant AEs were observed in the clinical study of the first-generation GD2-CAR (Pule et al., 2008; Louis et al., 2011). Compared to earlier generation receptors, the improved proliferation, CTL activity and cytokine secretion of the third-generation GD2-iCAR (Pule et al., 2005) may add to the risk of toxicity either from enhanced tumor lysis, or on-target, off-tumor effects on healthy tissues, and thus inclusion of iCasp9 offers the means to mitigate these risks.

CAR T cells have shown great initial promise in the clinic but this has been accompanied by some severe and even fatal side-effects. By significantly improving its safety profile, the iCasp9/AP1903 suicide gene technology can complement CAR T-cell technology and advance its more widespread adoption in the clinic.

## Conflict of Interest Statement

The authors declare that the research was conducted in the absence of any commercial or financial relationships that could be construed as a potential conflict of interest.

## References

[B1] Annunziata C. M. Kohn E. C. Lorusso P. Houston N. D. Coleman R. L. Buzoianu M. (2013). Phase 1, open-label study of MEDI-547 in patients with relapsed or refractory solid tumors. *Invest. New Drugs* 31 77–84. 10.1007/s10637-012-9801-2 22370972 PMC3553417

[B2] Berger C. Flowers M. E. Warren E. H. Riddell S. R. (2006). Analysis of transgene-specific immune responses that limit the in vivo persistence of adoptively transferred HSV-TK-modified donor T cells after allogeneic hematopoietic cell transplantation. *Blood* 107 2294–2302. 10.1182/blood-2005-08-3503 16282341 PMC1895724

[B3] Besser M. J. Shapira-Frommer R. Itzhaki O. Treves A. J. Zippel D. B. Levy D. (2013). Adoptive transfer of tumor-infiltrating lymphocytes in patients with metastatic melanoma: intent-to-treat analysis and efficacy after failure to prior immunotherapies. *Clin. Cancer Res.* 19 4792–4800. 10.1158/1078-0432.CCR-13-0380 23690483

[B4] Bonini C. Bondanza A. Perna S. K. Kaneko S. Traversari C. Ciceri F. (2007). The suicide gene therapy challenge: how to improve a successful gene therapy approach. *Mol. Ther.* 15 1248–1252. 10.1038/sj.mt.6300190 17505474

[B5] Brentjens R. J. Davila M. L. Riviere I. Park J. Wang X. Cowell L. G. (2013). CD19-targeted T cells rapidly induce molecular remissions in adults with chemotherapy-refractory acute lymphoblastic leukemia. *Sci. Transl. Med.* 5 177ra138. 10.1126/scitranslmed.3005930 PMC3742551 23515080

[B6] Brentjens R. J. Riviere I. Park J. H. Davila M. L. Wang X. Stefanski J. (2011). Safety and persistence of adoptively transferred autologous CD19-targeted T cells in patients with relapsed or chemotherapy refractory B-cell leukemias. *Blood* 118 4817–4828. 10.1182/blood-2011-04-348540 21849486 PMC3208293

[B7] Brentjens R. Yeh R. Bernal Y. Riviere I. Sadelain M. (2010). Treatment of chronic lymphocytic leukemia with genetically targeted autologous T cells: case report of an unforeseen adverse event in a phase I clinical trial. *Mol. Ther.* 18 666–668. 10.1038/mt.2010.31 20357779 PMC2862525

[B8] Budde L. E. Berger C. Lin Y. Wang J. Lin X. Frayo S. E. (2013). Combining a CD20 chimeric antigen receptor and an inducible caspase 9 suicide switch to improve the efficacy and safety of T cell adoptive immunotherapy for lymphoma. *PLoS ONE* 8:e82742. 10.1371/journal.pone.0082742 PMC3866194 24358223

[B9] Cattoglio C. Facchini G. Sartori D. Antonelli A. Miccio A. Cassani B. (2007). Hot spots of retroviral integration in human CD34^+^ hematopoietic cells. *Blood* 110 1770–1778. 10.1182/blood-2007-01-068759 17507662

[B10] Chien K. R. (2006). Herceptin and the heart–a molecular modifier of cardiac failure. *N. Engl. J. Med.* 354 789–790. 10.1056/NEJMp058315 16495390

[B11] Ciceri F. Bonini C. Stanghellini M. T. Bondanza A. Traversari C. Salomoni M. (2009). Infusion of suicide-gene-engineered donor lymphocytes after family haploidentical haemopoietic stem-cell transplantation for leukaemia (the TK007 trial): a non-randomised phase I-II study. *Lancet Oncol.* 10 489–500. 10.1016/S1470-2045(09)70074-9 19345145

[B12] Clackson T. Yang W. Rozamus L. W. Hatada M. Amara J. F. Rollins C. T. (1998). Redesigning an FKBP-ligand interface to generate chemical dimerizers with novel specificity. *Proc. Natl. Acad. Sci. U.S.A.* 95 10437–10442. 10.1073/pnas.95.18.10437 9724721 PMC27912

[B13] Cooper L. J. Ausubel L. Gutierrez M. Stephan S. Shakeley R. Olivares S. (2006). Manufacturing of gene-modified cytotoxic T lymphocytes for autologous cellular therapy for lymphoma. *Cytotherapy* 8 105–117. 10.1080/14653240600620176 16698684

[B14] de Witte M. A. Jorritsma A. Swart E. Straathof K. C. De Punder K. Haanen J. B. (2008). An inducible caspase 9 safety switch can halt cell therapy-induced autoimmune disease. *J. Immunol.* 180 6365–6373. 10.4049/jimmunol.180.9.6365 18424760

[B15] Di Stasi A. Tey S. K. Dotti G. Fujita Y. Kennedy-Nasser A. Martinez C. (2011). Inducible apoptosis as a safety switch for adoptive cell therapy. *N. Engl. J. Med.* 365 1673–1683. 10.1056/NEJMoa1106152 22047558 PMC3236370

[B16] Fridman W. H. Pages F. Sautes-Fridman C. Galon J. (2012). The immune contexture in human tumours: impact on clinical outcome. *Nat. Rev. Cancer* 12 298–306. 10.1038/nrc3245 22419253

[B76] Gargett T. Fraser C. K. Dotti G. Yvon E. S. Brown M. P. (2014). BRAF and MEK inhibition variably affect GD2-specific Chimeric Antigen Receptor (CAR) T cell function *in vitro*. *J. Immunother.* (in press). 10.1097/CJI.0000000000000061 25415284

[B17] Grupp S. A. Kalos M. Barrett D. Aplenc R. Porter D. L. Rheingold S. R. (2013). Chimeric antigen receptor-modified T cells for acute lymphoid leukemia. *N. Engl. J. Med.* 368 1509–1518. 10.1056/NEJMoa1215134 23527958 PMC4058440

[B18] Hacein-Bey-Abina S. Hauer J. Lim A. Picard C. Wang G. P. Berry C. C. (2010). Efficacy of gene therapy for X-linked severe combined immunodeficiency. *N. Engl. J. Med.* 363 355–364. 10.1056/NEJMoa1000164 20660403 PMC2957288

[B19] Hacein-Bey-Abina S. Von Kalle C. Schmidt M. Le Deist F. Wulffraat N. Mcintyre E. (2003). A serious adverse event after successful gene therapy for X-linked severe combined immunodeficiency. *N. Engl. J. Med.* 348 255–256. 10.1056/NEJM200301163480314 12529469

[B20] Hamid O. Robert C. Daud A. Hodi F. S. Hwu W. J. Kefford R. (2013). Safety and tumor responses with lambrolizumab (anti-PD-1) in melanoma. *N. Engl. J. Med.* 369 134–144. 10.1056/NEJMoa1305133 23724846 PMC4126516

[B21] Heslop H. E. Slobod K. S. Pule M. A. Hale G. A. Rousseau A. Smith C. A. (2010). Long-term outcome of EBV-specific T-cell infusions to prevent or treat EBV-related lymphoproliferative disease in transplant recipients. *Blood* 115 925–935. 10.1182/blood-2009-08-239186 19880495 PMC2817637

[B22] Hodi F. S. O’day S. J. Mcdermott D. F. Weber R. W. Sosman J. A. Haanen J. B. (2010). Improved survival with ipilimumab in patients with metastatic melanoma. *N. Engl. J. Med.* 363 711–723. 10.1056/NEJMoa1003466 20525992 PMC3549297

[B23] Hoyos V. Savoldo B. Quintarelli C. Mahendravada A. Zhang M. Vera J. (2010). Engineering CD19-specific T lymphocytes with interleukin-15 and a suicide gene to enhance their anti-lymphoma/leukemia effects and safety. *Leukemia* 24 1160–1170. 10.1038/leu.2010.75 20428207 PMC2888148

[B24] Iuliucci J. D. Oliver S. D. Morley S. Ward C. Ward J. Dalgarno D. (2001). Intravenous safety and pharmacokinetics of a novel dimerizer drug, AP1903, in healthy volunteers. *J. Clin. Pharmacol.* 41 870–879. 10.1177/00912700122010771 11504275

[B25] Jensen M. C. Popplewell L. Cooper L. J. Digiusto D. Kalos M. Ostberg J. R. (2010). Antitransgene rejection responses contribute to attenuated persistence of adoptively transferred CD20/CD19-specific chimeric antigen receptor redirected T cells in humans. *Biol. Blood Marrow Transplant.* 16 1245–1256. 10.1016/j.bbmt.2010.03.014 20304086 PMC3383803

[B26] Ji J. Mould D. R. Blum K. A. Ruppert A. S. Poi M. Zhao Y. (2013). A pharmacokinetic/pharmacodynamic model of tumor lysis syndrome in chronic lymphocytic leukemia patients treated with flavopiridol. *Clin. Cancer Res.* 19 1269–1280. 10.1158/1078-0432.CCR-12-1092 23300276 PMC3845832

[B27] Johnson L. A. Morgan R. A. Dudley M. E. Cassard L. Yang J. C. Hughes M. S. (2009). Gene therapy with human and mouse T-cell receptors mediates cancer regression and targets normal tissues expressing cognate antigen. *Blood* 114 535–546. 10.1182/blood-2009-03-211714 19451549 PMC2929689

[B28] Kalos M. Levine B. L. Porter D. L. Katz S. Grupp S. A. Bagg A. (2011). T cells with chimeric antigen receptors have potent antitumor effects and can establish memory in patients with advanced leukemia. *Sci. Transl. Med.* 3 95ra73. 10.1126/scitranslmed.3002842 PMC3393096 21832238

[B29] Kochenderfer J. N. Dudley M. E. Feldman S. A. Wilson W. H. Spaner D. E. Maric I. (2012). B-cell depletion and remissions of malignancy along with cytokine-associated toxicity in a clinical trial of anti-CD19 chimeric-antigen-receptor-transduced T cells. *Blood* 119 2709–2720. 10.1182/blood-2011-10-384388 22160384 PMC3327450

[B30] Lamers C. H. Sleijfer S. Van Steenbergen S. Van Elzakker P. Van Krimpen B. Groot C. (2013). Treatment of metastatic renal cell carcinoma with CAIX CAR-engineered T cells: clinical evaluation and management of on-target toxicity. *Mol. Ther.* 21 904–912. 10.1038/mt.2013.17 23423337 PMC5189272

[B31] Lamers C. H. Sleijfer S. Vulto A. G. Kruit W. H. Kliffen M. Debets R. (2006). Treatment of metastatic renal cell carcinoma with autologous T-lymphocytes genetically retargeted against carbonic anhydrase IX: first clinical experience. *J. Clin. Oncol.* 24 e20–e22. 10.1200/JCO.2006.05.9964 16648493

[B32] Linette G. P. Stadtmauer E. A. Maus M. V. Rapoport A. P. Levine B. L. Emery L. (2013). Cardiovascular toxicity and titin cross-reactivity of affinity-enhanced T cells in myeloma and melanoma. *Blood* 122 863–871. 10.1182/blood-2013-03-490565 23770775 PMC3743463

[B33] Louis C. U. Savoldo B. Dotti G. Pule M. Yvon E. Myers G. D. (2011). Antitumor activity and long-term fate of chimeric antigen receptor-positive T cells in patients with neuroblastoma. *Blood* 118 6050–6056. 10.1182/blood-2011-05-354449 21984804 PMC3234664

[B34] Marin V. Cribioli E. Philip B. Tettamanti S. Pizzitola I. Biondi A. (2012). Comparison of different suicide-gene strategies for the safety improvement of genetically manipulated T cells. *Hum. Gene Ther. Methods* 23 376–386. 10.1089/hgtb.2012.050 23186165 PMC4015080

[B35] Maude S. L. Barrett D. Teachey D. T. Grupp S. A. (2014). Managing cytokine release syndrome associated with novel T cell-engaging therapies. *Cancer J.* 20 119–122. 10.1097/PPO.0000000000000035 24667956 PMC4119809

[B36] Maus M. V. Grupp S. A. Porter D. L. June C. H. (2014). Antibody-modified T cells: CARs take the front seat for hematologic malignancies. *Blood* 123 2625–2635. 10.1182/blood-2013-11-492231 24578504 PMC3999751

[B37] Mocellin S. Pasquali S. Rossi C. R. Nitti D. (2010). Interferon alpha adjuvant therapy in patients with high-risk melanoma: a systematic review and meta-analysis. *J. Natl. Cancer Inst.* 102 493–501. 10.1093/jnci/djq009 20179267

[B38] Morgan R. A. Chinnasamy N. Abate-Daga D. Gros A. Robbins P. F. Zheng Z. (2013). Cancer regression and neurological toxicity following anti-MAGE-A3 TCR gene therapy. *J. Immunother.* 36 133–151. 10.1097/CJI.0b013e3182829903 23377668 PMC3581823

[B39] Morgan R. A. Yang J. C. Kitano M. Dudley M. E. Laurencot C. M. Rosenberg S. A. (2010). Case report of a serious adverse event following the administration of T cells transduced with a chimeric antigen receptor recognizing ERBB2. *Mol. Ther.* 18 843–851. 10.1038/mt.2010.24 20179677 PMC2862534

[B40] Murray J. L. Cunningham J. E. Brewer H. Mujoo K. Zukiwski A. A. Podoloff D. A. (1994). Phase I trial of murine monoclonal antibody 14G2a administered by prolonged intravenous infusion in patients with neuroectodermal tumors. *J. Clin. Oncol.* 12 184–193 8270976 10.1200/JCO.1994.12.1.184

[B41] Newrzela S. Al-Ghaili N. Heinrich T. Petkova M. Hartmann S. Rengstl B. (2012). T-cell receptor diversity prevents T-cell lymphoma development. *Leukemia* 26 2499–2507. 10.1038/leu.2012.142 22643706

[B42] Newrzela S. Cornils K. Heinrich T. Schlager J. Yi J. H. Lysenko O. (2011). Retroviral insertional mutagenesis can contribute to immortalization of mature T lymphocytes. *Mol. Med.* 17 1223–1232. 10.2119/molmed.2010.00193 PMC3321800 21826372

[B43] Newrzela S. Cornils K. Li Z. Baum C. Brugman M. H. Hartmann M. (2008). Resistance of mature T cells to oncogene transformation. *Blood* 112 2278–2286. 10.1182/blood-2007-12-128751 18566328

[B44] Park J. H. Brentjens R. J. (2010). Adoptive immunotherapy for B-cell malignancies with autologous chimeric antigen receptor modified tumor targeted T cells. *Discov. Med.* 9 277–288 20423671 PMC4697441

[B45] Parkhurst M. R. Yang J. C. Langan R. C. Dudley M. E. Nathan D. A. Feldman S. A. (2011). T cells targeting carcinoembryonic antigen can mediate regression of metastatic colorectal cancer but induce severe transient colitis. *Mol. Ther.* 19 620–626. 10.1038/mt.2010.272 21157437 PMC3048186

[B46] Perez-Soler R. Delord J. P. Halpern A. Kelly K. Krueger J. Sureda B. M. (2005). HER1/EGFR inhibitor-associated rash: future directions for management and investigation outcomes from the HER1/EGFR inhibitor rash management forum. *Oncologist* 10 345–356. 10.1634/theoncologist.10-5-345 15851793

[B47] Porter D. L. Levine B. L. Kalos M. Bagg A. June C. H. (2011). Chimeric antigen receptor-modified T cells in chronic lymphoid leukemia. *N. Engl. J. Med.* 365 725–733. 10.1056/NEJMoa1103849 21830940 PMC3387277

[B48] Pule M. A. Savoldo B. Myers G. D. Rossig C. Russell H. V. Dotti G. (2008). Virus-specific T cells engineered to coexpress tumor-specific receptors: persistence and antitumor activity in individuals with neuroblastoma. *Nat. Med.* 14 1264–1270. 10.1038/nm.1882 18978797 PMC2749734

[B49] Pule M. A. Straathof K. C. Dotti G. Heslop H. E. Rooney C. M. Brenner M. K. (2005). A chimeric T cell antigen receptor that augments cytokine release and supports clonal expansion of primary human T cells. *Mol. Ther.* 12 933–941. 10.1016/j.ymthe.2005.04.016 15979412

[B50] Quintarelli C. Vera J. F. Savoldo B. Giordano Attianese G. M. Pule M. Foster A. E. (2007). Co-expression of cytokine and suicide genes to enhance the activity and safety of tumor-specific cytotoxic T lymphocytes. *Blood* 110 2793–2802. 10.1182/blood-2007-02-072843 17638856 PMC2018664

[B51] Recchia A. Bonini C. Magnani Z. Urbinati F. Sartori D. Muraro S. (2006). Retroviral vector integration deregulates gene expression but has no consequence on the biology and function of transplanted T cells. *Proc. Natl. Acad. Sci. U.S.A.* 103 1457–1462. 10.1073/pnas.0507496103 16432223 PMC1360534

[B52] Restifo N. P. Dudley M. E. Rosenberg S. A. (2012). Adoptive immunotherapy for cancer: harnessing the T cell response. *Nat. Rev. Immunol.* 12 269–281. 10.1038/nri3191 22437939 PMC6292222

[B53] Riddell S. R. Elliott M. Lewinsohn D. A. Gilbert M. J. Wilson L. Manley S. A. (1996). T-cell mediated rejection of gene-modified HIV-specific cytotoxic T lymphocytes in HIV-infected patients. *Nat. Med.* 2 216–223. 10.1038/nm0296-216 8574968

[B54] Rosenberg S. A. (2014). IL-2: the first effective immunotherapy for human cancer. *J. Immunol.* 192 5451–5458. 10.4049/jimmunol.1490019 24907378 PMC6293462

[B55] Rosenberg S. A. Dudley M. E. (2004). Cancer regression in patients with metastatic melanoma after the transfer of autologous antitumor lymphocytes. *Proc. Natl. Acad. Sci. U.S.A.* 101(Suppl. 2) 14639–14645. 10.1073/pnas.0405730101 15381769 PMC521998

[B56] Saleh M. N. Khazaeli M. B. Wheeler R. H. Dropcho E. Liu T. Urist M. (1992). Phase I trial of the murine monoclonal anti-GD2 antibody 14G2a in metastatic melanoma. *Cancer Res.* 52 4342–4347 1643631

[B57] Sangiolo D. Leuci V. Gallo S. Aglietta M. Piacibello W. (2011). Gene-modified T lymphocytes in the setting of hematopoietic cell transplantation: potential benefits and possible risks. *Expert Opin. Biol. Ther.* 11 655–666. 10.1517/14712598.2011.565325 21375466

[B58] Savoldo B. Ramos C. A. Liu E. Mims M. P. Keating M. J. Carrum G. (2011). CD28 costimulation improves expansion and persistence of chimeric antigen receptor-modified T cells in lymphoma patients. *J. Clin. Invest.* 121 1822–1826. 10.1172/JCI46110 21540550 PMC3083795

[B59] Scholler J. Brady T. L. Binder-Scholl G. Hwang W. T. Plesa G. Hege K. M. (2012). Decade-long safety and function of retroviral-modified chimeric antigen receptor T cells. *Sci. Transl. Med.* 4 132ra153. 10.1126/scitranslmed.3003761 PMC4368443 22553251

[B60] Seliger B. (2008). Molecular mechanisms of MHC class I abnormalities and APM components in human tumors. *Cancer Immunol. Immunother.* 57 1719–1726. 10.1007/s00262-008-0515-4 18408926 PMC11030176

[B61] Seliger B. Harders C. Wollscheid U. Staege M. S. Reske-Kunz A. B. Huber C. (1996). Suppression of MHC class I antigens in oncogenic transformants: association with decreased recognition by cytotoxic T lymphocytes. *Exp. Hematol.* 24 1275–1279 8862437

[B62] Shankaran V. Ikeda H. Bruce A. T. White J. M. Swanson P. E. Old L. J. (2001). IFNgamma and lymphocytes prevent primary tumour development and shape tumour immunogenicity. *Nature* 410 1107–1111. 10.1038/35074122 11323675

[B63] Sorkin L. S. Otto M. Baldwin W. M.3rd Vail E. Gillies S. D. Handgretinger R. (2010). Anti-GD(2) with an FC point mutation reduces complement fixation and decreases antibody-induced allodynia. *Pain* 149 135–142. 10.1016/j.pain.2010.01.024 20171010 PMC3755890

[B64] Straathof K. C. Pule M. A. Yotnda P. Dotti G. Vanin E. F. Brenner M. K. (2005). An inducible caspase 9 safety switch for T-cell therapy. *Blood* 105 4247–4254. 10.1182/blood-2004-11-4564 15728125 PMC1895037

[B65] Suntharalingam G. Perry M. R. Ward S. Brett S. J. Castello-Cortes A. Brunner M. D. (2006). Cytokine storm in a phase 1 trial of the anti-CD28 monoclonal antibody TGN1412. *N. Engl. J. Med.* 355 1018–1028. 10.1056/NEJMoa063842 16908486

[B66] Teachey D. T. Rheingold S. R. Maude S. L. Zugmaier G. Barrett D. M. Seif A. E. (2013). Cytokine release syndrome after blinatumomab treatment related to abnormal macrophage activation and ameliorated with cytokine-directed therapy. *Blood* 121 5154–5157. 10.1182/blood-2013-02-485623 23678006 PMC4123427

[B67] Tey S. K. Dotti G. Rooney C. M. Heslop H. E. Brenner M. K. (2007). Inducible caspase 9 suicide gene to improve the safety of allodepleted T cells after haploidentical stem cell transplantation. *Biol. Blood Marrow Transplant.* 13 913–924. 10.1016/j.bbmt.2007.04.005 17640595 PMC2040267

[B68] Topalian S. L. Sznol M. Mcdermott D. F. Kluger H. M. Carvajal R. D. Sharfman W. H. (2014). Survival, durable tumor remission, and long-term safety in patients with advanced melanoma receiving nivolumab. *J. Clin. Oncol.* 32 1020–1030. 10.1200/JCO.2013.53.0105 24590637 PMC4811023

[B69] Wang X. Naranjo A. Brown C. E. Bautista C. Wong C. W. Chang W. C. (2012). Phenotypic and functional attributes of lentivirus-modified CD19-specific human CD8^+^ central memory T cells manufactured at clinical scale. *J. Immunother.* 35 689–701. 10.1097/CJI.0b013e318270dec7 23090078 PMC3525345

[B70] Willmon C. L. Krabbenhoft E. Black M. E. (2006). A guanylate kinase/HSV-1 thymidine kinase fusion protein enhances prodrug-mediated cell killing. *Gene Ther.* 13 1309–1312. 10.1038/sj.gt.3302794 16810197

[B71] Wolchok J. D. Weber J. S. Maio M. Neyns B. Harmankaya K. Chin K. (2013). Four-year survival rates for patients with metastatic melanoma who received ipilimumab in phase II clinical trials. *Ann. Oncol.* 24 2174–2180. 10.1093/annonc/mdt161 23666915 PMC4081656

[B72] Wu X. Li Y. Crise B. Burgess S. M. (2003). Transcription start regions in the human genome are favored targets for MLV integration. *Science* 300 1749–1751. 10.1126/science.1083413 12805549

[B73] Yang H. Rosove M. H. Figlin R. A. (1999). Tumor lysis syndrome occurring after the administration of rituximab in lymphoproliferative disorders: high-grade non-Hodgkin’s lymphoma and chronic lymphocytic leukemia. *Am. J. Hematol.* 62 247–250. 10.1002/(SICI)1096-8652(199912)62:4<247::AID-AJH9>3.0.CO;2-T 10589082

[B74] Yu A. L. Gilman A. L. Ozkaynak M. F. London W. B. Kreissman S. G. Chen H. X. (2010). Anti-GD2 antibody with GM-CSF, interleukin-2, and isotretinoin for neuroblastoma. *N. Engl. J. Med.* 363 1324–1334. 10.1056/NEJMoa0911123 20879881 PMC3086629

[B75] Zhou X. Di Stasi A. Tey S. K. Krance R. A. Martinez C. Leung K. S. (2014). Long-term outcome after haploidentical stem cell transplant and infusion of T cells expressing the inducible caspase 9 safety transgene. *Blood* 123 3895–3905. 10.1182/blood-2014-01-551671 24753538 PMC4064331

